# Interim estimates of 2018/19 vaccine effectiveness against influenza A(H1N1)pdm09, Canada, January 2019

**DOI:** 10.2807/1560-7917.ES.2019.24.4.1900055

**Published:** 2019-01-24

**Authors:** Danuta M Skowronski, Siobhan Leir, Suzana Sabaiduc, Michelle Murti, James A Dickinson, Romy Olsha, Jonathan B Gubbay, Matthew A Croxen, Hugues Charest, Tracy Chan, Nathalie Bastien, Yan Li, Mel Krajden, Gaston De Serres

**Affiliations:** 1 British Columbia Centre for Disease Control, Vancouver, Canada; 2 University of British Columbia, Vancouver, Canada; 3 Public Health Ontario, Toronto, Canada; 4 University of Toronto, Toronto, Canada; 5 University of Calgary, Calgary, Canada; 6 Provincial Laboratory for Public Health, Edmonton, Canada; 7 University of Alberta, Edmonton, Canada; 8 Institut National de Santé Publique du Québec, Quebec City, Canada; 9 National Microbiology Laboratory, Public Health Agency of Canada, Winnipeg, Canada; 10 Laval University, Quebec City, Canada; 11 Centre Hospitalier Universitaire de Québec, Quebec City, Canada

**Keywords:** influenza, influenza virus, vaccine-preventable diseases, vaccines, immunisation, vaccine effectiveness, genomics, Canada, viral infections, influenza-like illness, ILI, epidemiology, laboratory

## Abstract

Using a test-negative design, the Canadian Sentinel Practitioner Surveillance Network assessed interim 2018/19 vaccine effectiveness (VE) against predominant influenza A(H1N1)pdm09 viruses. Adjusted VE was 72% (95% confidence interval: 60 to 81) against medically attended, laboratory-confirmed influenza A(H1N1)pdm09 illness. This substantial vaccine protection was observed in all age groups, notably young children who appeared to be disproportionately affected. Sequence analysis identified heterogeneity in emerging clade 6B.1 viruses but no dominant drift variant.

The 2018/19 influenza season in Canada for the period spanning November through January has been characterised by dominant influenza A(H1N1)pdm09 activity, with lesser influenza A(H3N2) and little influenza B contribution [1]. This profile is in contrast to the 2017/18 season which was comprised of dominant influenza A(H3N2) and early influenza B(Yamagata) co-circulation [2]. The last influenza A(H1N1)pdm09-dominant epidemics in Canada were in 2013/14 and 2015/16 [3,4].

The 2018/19 influenza vaccine for the northern hemisphere contains an A/Michigan/45/2015 (H1N1)pdm09-like antigen (belonging to clade 6B.1). The same component was included in the 2017/18 northern and the 2018 southern hemisphere vaccines [5]. Preliminary estimates of vaccine effectiveness (VE) from Australia’s 2018 season showed substantial VE of 78% (95% confidence interval (CI): 51 to 91) against influenza A(H1N1)pdm09 viruses [6]. Here we present interim 2018/19 VE estimates against influenza A(H1N1)pdm09 viruses from the Canadian Sentinel Practitioner Surveillance Network (SPSN), including detailed genetic characterisation of contributing viruses.

## Vaccine effectiveness evaluation

VE was estimated using a test-negative design, as previously described [2-4]. Nasal/nasopharyngeal specimens and epidemiological data were collected from patients presenting to community-based sentinel practitioners in Alberta, British Columbia, Ontario, and Quebec. Influenza-like illness (ILI) was defined as acute onset of self-reported fever and cough and at least one other symptom including sore throat, myalgia, arthralgia or prostration. Fever was not a requirement for elderly adults 65 years and older. Analyses were restricted to patients at least 1-year-old presenting within 7 days of ILI onset. Vaccination status was based on self-report of 2018/19 vaccine receipt at least 2 weeks before symptom onset; patients vaccinated less than 2 weeks before onset or with unknown vaccination status or timing were excluded. Compliance with two-dose recommendations in young children was not assessed. All influenza vaccines manufactured for Canada (including SPSN provinces) for 2018/19 were egg-based and the vast majority (> 95%) were inactivated vaccines (i.e. the live attenuated influenza vaccine constituted < 5% of doses distributed by the publicly-funded immunisation campaign). A high dose inactivated formulation was available for elderly adults in the SPSN province of Ontario. Institutional review boards in each province provided ethical approval.

Specimens collected from week 45 (starting 4 November 2018) to week 2 (ending 12 January 2019) were tested for influenza type and subtype by real-time RT-PCR assays at provincial public health reference laboratories. Sanger sequencing of the haemagglutinin (HA) gene (HA1 and HA2) of A(H1N1)pdm09 viruses contributing to VE analyses was conducted for all SPSN provinces at the British Columbia Centre for Disease Control Public Health Laboratory. Virus sequencing was undertaken on original patient specimens, including as many as possible in the order received. Viral sequence data are being deposited for reference into the Global Initiative on Sharing All Influenza Data (GISAID) platform (www.gisaid.org )

Emerging genetic variants were assigned to genetic subgroups based on defining amino acid substitutions [7,8]. Substitutions were assessed for their involvement of HA1 antigenic sites (especially immuno-dominant Sa and Sb, but also Ca1, Ca2 or Cb), or as otherwise relevant to diversifying selection [9-11]. Sequence analysis was in relation to the cell-passaged A/Michigan/45/2015 vaccine reference strain and the corresponding egg-adapted version (A/Michigan/45/2015 X-275) as well as an alternate egg-adapted strain (A/Singapore/GP1908/2015 IVR-180) also used by manufacturers.

Odds ratios (OR) comparing influenza test positivity between vaccinated and unvaccinated participants were calculated using a logistic regression model, adjusted for age group, province, time from ILI onset to specimen collection, and specimen collection date. VE was derived as (1 − OR) × 100%. VE was estimated against influenza A(H1N1)pdm09 in the primary analysis, but estimates against any influenza and influenza A are also presented.

## Epidemiological findings

**Figure 1:**
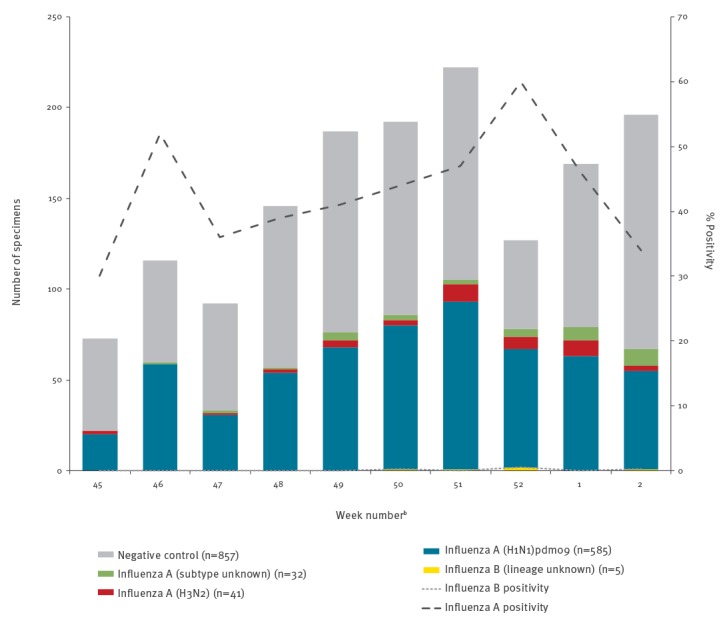
Influenza detections among eligible patients presenting with influenza-like illness, by week of specimen collection, Canadian Sentinel Practitioner Surveillance Network, 4 November 2018–12 January 2019 (n = 1,518)^a^

^a^ One specimen was co-infected with influenza A(H1N1)pdm09 and influenza B and another specimen was co-infected with influenza A(H1N1)pdm09 and A(H3N2). Viruses involved in co-infections are plotted separately by type/subtype such that specimens involved in co-infections appear twice.

^b^ Missing specimen collection dates were imputed as the date the specimen was received and processed at the provincial laboratory minus 2 days, the average time between specimen collection date and laboratory received date among specimens with complete information for both values. Data for week 2 may be incomplete given delays in specimen processing and laboratory testing.

Among 1,518 eligible specimens, 661 (44%) tested positive for influenza, including 656 (99%) influenza A, 4 (1%) influenza B and 1 (0.2%) influenza A/B co-infection. Among the 626 (95%) subtyped influenza A viruses, 585 (93%) were A(H1N1)pdm09 and 41 (7%) were A(H3N2) (Figure 1). There were two co-infections involving influenza A(H1N1)pdm09: one with influenza B and another with influenza A(H3N2). 

Participant profiles are displayed in Table 1 for the 585 influenza A(H1N1)pdm09 cases and 857 test-negative controls included in primary VE analysis. Most (62%) participants were adults 20–64-years-old. However, significantly more influenza A(H1N1)pdm09 cases (28%) than controls (14%) were children 1–8-years-old (p < 0.001); that age group also had the highest influenza A(H1N1)pdm09 test positivity (58%; 163/282). Conversely, significantly fewer influenza A(H1N1)pdm09 cases than controls were 65 years or older (4% vs 13%; p < 0.001), and this age group exhibited the lowest influenza A(H1N1)pdm09 test positivity (18%; 25/138). Overall, 27% of controls but just 8% of influenza A(H1N1)pdm09 cases were considered vaccinated (p < 0.001) (Table 1).

**Table 1:** Participant profile, by influenza A(H1N1)pdm09 case and vaccination status, Canadian Sentinel Practitioner Surveillance Network, 4 November 2018–12 January 2019 (n = 1,442)

Characteristic	Overall		Distribution by case status (column %)					Vaccination coverage (row %)^b^					
			A(H1N1)pdm09 cases		Negative controls		p value^a^	A(H1N1)pdm09 cases		p value^c^	Negative controls		p value^c^
	n	%	n	%	n	%		n	%		n	%	
n (row %)	1,442	100	585	41	857	59	NA	45	8	NA	234	27	NA
**Age group (years)**													
1–8	282	20	163	28	119	14	< 0.001	3	2	< 0.001	21	18	< 0.001
9–19	134	9	54	9	80	9		2	4		9	11	
20–49	632	44	253	43	379	44		19	8		78	21	
50–64	256	18	90	15	166	19		12	13		55	33	
≥ 65	138	10	25	4	113	13		9	36		71	63	
Median age (range)	35 (1–97)		31 (1–82)		38 (1–97)		< 0.001	48 (2–82)		< 0.001	51.5 (1–97)		< 0.001
**Sex**													
Female	869	60	328	56	541	63	0.005	29	9	0.25	170	31	< 0.001
Male	564	39	255	44	309	36		16	6		61	20	
Unknown	9	1	2	0	7	1	NA	0	0	NA	3	43	NA
**Comorbidity^d^**													
No	1,099	76	474	81	625	73	< 0.001	30	6	0.01	141	23	< 0.001
Yes	264	18	76	13	188	22		11	14		85	45	
Unknown	79	5	35	6	44	5	NA	4	11	NA	8	18	NA
**Province**													
Alberta	432	30	219	37	213	25	< 0.001	16	7	0.02	65	31	0.08
British Columbia	267	19	87	15	180	21		10	11		55	31	
Ontario	546	38	179	31	367	43		18	10		97	26	
Quebec	197	14	100	17	97	11		1	1		17	18	
**Specimen collection interval from ILI onset (days)^e^**													
≤ 4	1,066	74	476	81	590	69	< 0.001	38	8	0.58	150	25	0.07
5–7	376	26	109	19	267	31		7	6		84	31	
Median interval (range)	3 (0–7)		3 (0–7)		3 (0–7)		< 0.001	3 (0–7)		0.61	4 (0–7)		0.04
**Month of specimen collection**													
November	409	28	158	27	251	29	0.006	7	4	0.14	51	20	0.01
December	736	51	326	56	410	48		27	8		121	30	
January	297	21	101	17	196	23		11	11		62	32	
**Vaccination status **													
Vaccination without regard to timing^f^	334/ 1,497	22	62/ 602	10	272/ 895	30	< 0.001	NA	NA	NA	NA	NA	NA
≥ 2 weeks before ILI onset	279	19	45	8	234	27	< 0.001	NA	NA	NA	NA	NA	NA

ILI: influenza-like illness; NA: not applicable.

Unless otherwise specified, values displayed in the columns represent the number of specimens per category and percentages are relative to the total. Where the denominator for the percentages differs from the total, fractions supporting the calculation of percentages are shown.

^a^ p values for comparison between cases and controls were derived by chi-squared test, Fisher’s exact test or Wilcoxon rank-sum test.

^b^ Vaccination status based on patients’ self-report; defined as receipt of 2018/19 seasonal influenza vaccine at least 2 weeks before symptom onset. Patients vaccinated less than 2 weeks before onset of symptoms or with unknown vaccination status or timing were excluded.

^c^ p values for comparison of the proportion vaccinated were derived by chi-squared test, Fisher’s exact test or Wilcoxon rank-sum test.

^d^ Includes chronic comorbidities that place individuals at higher risk of serious complications from influenza as defined by Canada’s National Advisory Committee on Immunization, including: heart, pulmonary (including asthma), renal, metabolic (such as diabetes), blood, cancer or immunocompromising conditions, conditions that compromise management of respiratory secretions and increase risk of aspiration, or morbid obesity (body mass index ≥ 40).

^e^ Missing specimen collection dates were imputed as the date the specimen was received and processed at the laboratory minus 2 days, the average time between specimen collection date and laboratory received date among specimens with complete information for both values.

^f^ Participants who received seasonal 2018/19 influenza vaccine less than 2 weeks before ILI onset or for whom vaccination timing was unknown were excluded from the primary analysis. They are included here for assessing vaccination regardless of timing for comparison to other estimates of vaccination coverage.

After adjustment for relevant covariates, VE against any influenza, foremost driven by A(H1N1)pdm09 viruses, was 68% (95% CI: 55 to 77); for influenza A(H1N1)pdm09 alone, it was 72% (95% CI: 60 to 81) (Table 2). Estimates for influenza A(H1N1)pdm09 were similar in sensitivity analyses: with additional adjustment for sex and comorbidity, VE was 74% (95% CI: 61 to 82), and with restriction to specimens collected from 2 December 2018 (week 49), VE was 70% (95% CI: 55 to 8). By age group, adjusted VE estimates against influenza A(H1N1)pdm09 were: 91% (95% CI: 67 to 98) in 1–8-year-old children, 71% (95% CI: −60 to 95) in 9–19-year-old children, 68% (95% CI: 51 to 80) in 20–64-year-old adults and 65% (95% CI: −1 to 88) in adults 65 years and older.

**Table 2:** Interim vaccine effectiveness estimates against any influenza infection, influenza A, and influenza A(H1N1)pdm09, Canadian Sentinel Practitioner Surveillance Network, 4 November 2018–12 January 2019 (n = 1,518)

Model	Any influenza		Influenza A		Influenza A(H1N1)pdm09	
Primary analysis – all participants	n vac/N	%	n vac/N	%	n vac/N	%
Total	1,518		1,514		1,442	
Cases	59/661	9	58/657	9	45/585	8
Controls	234/857	27	234/857	27	234/857	27
Vaccine effectiveness	%	95% CI	%	95% CI	%	95% CI
Unadjusted	74	65 to 81	74	65 to 81	78	69 to 84
Univariate adjustment for						
- Age group (1–8, 9–19, 20–49, 50–64, ≥ 65 years)	69	57 to 77	69	57 to 78	73	61 to 81
- Province (AB, BC, ON, QC)	73	63 to 80	73	63 to 80	77	68 to 84
- Interval from ILI onset to specimen collection (≤ 4, 5–7 days)	73	63 to 80	73	64 to 80	77	68 to 84
- Calendar time^a^	75	66 to 81	75	66 to 82	78	69 to 84
Full covariate adjustment^b^	68	55 to 77	68	55 to 77	72	60 to 81
Age-restricted analyses						
Participants 1–8 years-old	n vac/N	%	n vac/N	%	n vac/N	%
Total	289		289		282	
Cases	4/170	2	4/170	2	3/163	2
Controls	21/119	18	21/119	18	21/119	18
Vaccine effectiveness	%	95% CI	%	95% CI	%	95% CI
Unadjusted	89	66 to 96	89	66 to 96	91	70 to 97
Full covariate adjustment^c^	88	60 to 96	88	60 to 96	91	67 to 98
Participants 9–19 years-old	n vac/N	%	n vac/N	%	n vac/N	%
Total	138		138		134	
Cases	2/58	3	2/58	3	2/54	4
Controls	9/80	11	9/80	11	9/80	11
Vaccine effectiveness	%	95% CI	%	95% CI	%	95% CI
Unadjusted	72	−36 to 94	72	−36 to 94	70	−46 to 94
Full covariate adjustment^c^	71	−56 to 95	71	−56 to 95	71	−60 to 95
Participants 20–64 years-old	n vac/N	%	n vac/N	%	n vac/N	%
Total	946		943		888	
Cases	41/401	10	40/398	10	31/343	9
Controls	133/545	24	133/545	24	133/545	24
Vaccine effectiveness	%	95% CI	%	95% CI	%	95% CI
Unadjusted	65	49 to 76	65	49 to 76	69	53 to 80
Full covariate adjustment^d^	63	46 to 75	64	46 to 76	68	51 to 80
Participants ≥ 65 years-old	n vac/N	%	n vac/N	%	n vac/N	%
Total	145		144		138	
Cases	12/32	38	12/31	39	9/25	36
Controls	71/113	63	71/113	63	71/113	63
Vaccine effectiveness	%	95% CI	%	95% CI	%	95% CI
Unadjusted	65	20 to 84	63	15 to 83	67	18 to 86
Full covariate adjustment^c^	64	8 to 86	63	5 to 85	65	−1 to 88

AB: Alberta; BC: British Columbia; CI: confidence interval; ILI: influenza-like illness; n vac: number vaccinated; ON: Ontario; QC: Quebec.

^a^ Calendar time is based on week of specimen collection, modelled using cubic spline function with three equally spaced knots.

^b^ Age group (1–8, 9–19, 20–49, 50–64, ≥ 65 years), province, specimen collection interval and calendar time.

^c^ Province, specimen collection interval and calendar time.

^d^ Age group (20–49, 50–64 years), province, specimen collection interval and calendar time.

## Virological findings

**Figure 2:**
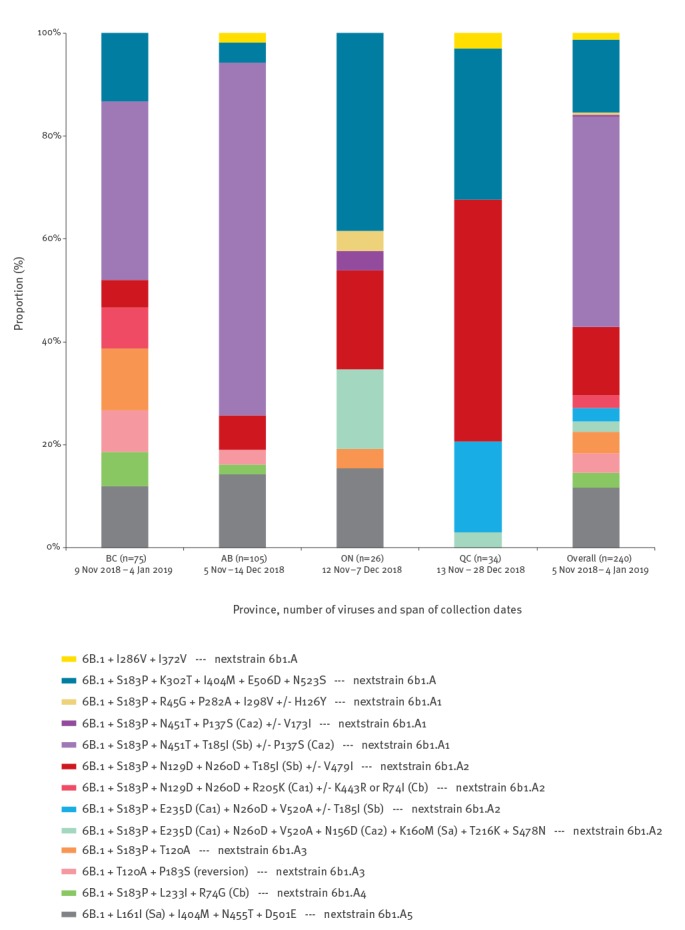
Distribution of clade 6B.1 variants by province, Canadian Sentinel Practitioner Surveillance Network, 5 November 2018–4 January 2019 (n = 240)

AB: Alberta; BC: British Columbia; ON: Ontario; QC: Quebec.

Sequencing of the HA gene was available for 240 (41%) of 585 influenza A(H1N1)pdm09 viruses. Collection dates of sequenced viruses spanned from 5 November 2018 to 4 January 2019 (Table 3, Figure 2). All sequenced viruses belonged to genetic clade 6B.1, to which the A/Michigan/45/2015 vaccine reference virus also belongs. However, all sequenced viruses additionally bore substitutions S74R (Cb), S164T (Sa) and I295V compared with the cell-passaged A/Michigan/45/2015 vaccine strain (except for nine viruses which showed continued drift at position 74). All viruses also bore additional substitutions M209K and R223K (receptor-binding site) attributed to egg adaptation mutations in the A/Michigan/45/2015 X-275 vaccine strain. 

**Table 3:** Virological profile of influenza A(H1N1)pdm09 specimens contributing to interim 2018/19 vaccine effectiveness evaluation, Canadian Sentinel Practitioner Surveillance Network, 5 November 2018–4 January 2019 (n = 240)

Nextstrain subgroup [8]	Genetic clade^a^ with subgroup substitutions^b^	British Columbia^c^		Alberta^d^		Ontario^e^		Quebec^f^		Overall^g^	
		n	%	n	%	n	%	n	%	n	%
6b1.A	6B.1 + I286V + I372V	0	0	2	2	0	0	1	3	3	1
	6B.1 + S183P + K302T + I404M + E506D + N523S	10	13	4	4	10	38	10	29	34	14
6b1.A1	6B.1 + S183P + R45G + P282A + I298V + H126Y	0	0	0	0	1	4	0	0	1	0
	6B.1 + S183P + N451T + **P137S (Ca2)**	0	0	0	0	1	4	0	0	1	0
	6B.1 + S183P + N451T + **T185I (Sb)**	26	35	72	69	0	0	0	0	98	41
6b1.A2	6B.1 + S183P + N129D + N260D + **T185I (Sb)**	2	3	7	7	5	19	16	47	30	13
	6B.1 + S183P + N129D + N260D + **T185I (Sb)** + V479I	2	3	0	0	0	0	0	0	2	1
	6B.1 + S183P + N129D + N260D + **R205K (Ca1)** + K443R	4	5	0	0	0	0	0	0	4	2
	6B.1 + S183P + N129D + N260D + **R205K (Ca1)** + **R74I (Cb)**	2	3	0	0	0	0	0	0	2	1
	6B.1 + S183P + **E235D (Ca1)** + N260D + V520A + **T185I (Sb)**	0	0	0	0	0	0	6	18	6	3
	6B.1 + S183P + **E235D (Ca1)** + N260D + V520A + **N156D (Ca2)** + **K160M (Sa)** + T216K + S478N	0	0	0	0	4	15	1	3	5	2
6b1.A3	6B.1 + S183P + T120A	9	12	0	0	1	4	0	0	10	4
	6B.1 + T120A + P183S (reversion)	6	8	3	3	0	0	0	0	9	4
6b1.A4	6B.1 + S183P + L233I + **R74G (Cb)**	5	7	2	2	0	0	0	0	7	3
6b1.A5	6B.1 + **L161I (Sa)** + I404M + N455T + D501E	9	12	15	14	4	15	0	0	28	12
**Total A(H1N1)pdm09 viruses sequenced**		75	100	105	100	26	100	34	100	240	100

^a^ Unless otherwise stated in the subgroup substitutions, all 6B.1 viruses also bear the substitutions S74R (Cb), S164T (Sa) and I295V relative to the cell-passaged vaccine strain A/Michigan/45/2015 plus M209K and R223K (receptor-binding site) relative to the egg-adapted version (X-275). Compared to the alternate egg-passaged vaccine strain (A/Singapore/GP1908/2015 IVR-180), viruses additionally bore T120A (except in those subgroups already bearing T120A)​, plus M209K and A225G (receptor-binding site), the latter instead of R223K.

^b^ Antigenic site substitutions are shown in bold, and the antigenic site follows in brackets. H1 numbering is based on influenza A(H1N1)pdm09 with the signal peptide removed. Genetic variants displayed here have been aligned with nextstrain subgrouping [8], recognising differences in numbering approaches (HA1 and HA2).

^c^ HA sequences available for 75 of 87 (86%) A(H1N1)pdm09 viruses contributing to analyses, with specimen collection dates spanning 9 November 2018–4 January 2019.

^d^ HA sequences available for 105 of 219 (48%) A(H1N1)pdm09 viruses contributing to analyses with specimen collection dates spanning 5 November–14 December 2018.

^e^ HA sequences available for 26 of 179 (15%) A(H1N1)pdm09 viruses contributing to analyses with specimen collection dates spanning 12 November–7 December 2018.

^f^ HA sequences available for 34 of 100 (34%) A(H1N1)pdm09 viruses contributing to analyses with specimen collection dates spanning 13 November–28 December 2018.

^g^ HA sequences available for 240 of 585 (41%) A(H1N1)pdm09 viruses contributing to analyses with specimen collection dates spanning 5 November 2018–4 January 2019.

Based on Sanger sequencing of the haemagglutinin (HA) gene. H1 numbering is based on influenza A(H1N1)pdm09 with the signal peptide removed. Genetic variants displayed here have been aligned with nextstrain subgrouping [8], recognising differences in numbering approaches (HA1 and HA2).

Unless otherwise stated in the subgroup substitutions, all 6B.1 viruses also bear the substitutions S74R (Cb), S164T (Sa) and I295V relative to the cell-passaged vaccine strain A/Michigan/45/2015 plus M209K and R223K (receptor-binding site) relative to the egg-adapted version (X-275). Compared to the alternate egg-passaged vaccine strain (A/Singapore/GP1908/2015 IVR-180), viruses additionally bore T120A (except in those subgroups already bearing T120A)​, plus M209K and A225G (receptor-binding site), the latter instead of R223K.

Beyond these shared substitutions, we observed heterogeneity among sequenced influenza A(H1N1)pdm09 viruses overall and by province, with no single subgroup dominating. Across subgroups, an S183P (non-antigenic site) substitution was found in 200 (83%) of 240 viruses and T185I (Sb) was found in 136 (57%) of 240 viruses. In Alberta, where there was an earlier epidemic peak [1], most viruses (72/105; 69%) belonged to a 6B.1 subgroup bearing T185I (Sb) with S183P and N451T substitutions (both non-antigenic sites); the same variant was also identified in a substantial proportion of viruses in British Columbia (26/75; 35%). In Ontario and Quebec, where fewer viruses contributed to sequence analysis, a different mix of subgroups was identified (Table 3, Figure 2).

## Discussion

In this interim analysis, the 2018/19 influenza vaccine is estimated to have reduced the risk of medically attended influenza A(H1N1)pdm09 illness in Canada by 72%. This 2018/19 mid-season VE estimate against dominant influenza A(H1N1)pdm09 viruses is substantially higher than last reported in the mid-season analysis from Canada for the 2017/18 A(H3N2)-dominant influenza epidemic, for which VE against A(H3N2) viruses was below 20% (with a paucity of A(H1N1)pdm09 cases detected) [2].

Our 2018/19 VE estimate of 72% (95% CI: 60 to 81) against influenza A(H1N1)pdm09 viruses is comparable to a preliminary report from Australia using the same vaccine component for their 2018 season (78%) [6]. Both estimates are higher than reported in prior meta-analysis for influenza A(H1N1)pdm09 viruses (61%; 95% CI: 57 to 65) [12]. The Canadian SPSN estimate for 2018/19 is similar to mid-season estimates from our network during the last two A(H1N1)pdm09-dominant epidemics in 2013/14 and 2015/16 [3,4]. Of note, the 2013/14 epidemic peaked in January 2014, with comparable VE estimates at mid- and end-of-season analysis (74%; 95% CI: 58 to 83 and 71%; 95% CI: 58 to 80, respectively) [10]. Conversely, the mid-season VE estimate for 2015/16 was substantially higher than the end-of-season estimate (64%; 95% CI: 44 to 77 vs 43%; 95% CI: 25 to 57), a finding that may in part be explained by waning of immunity and the unusually delayed epidemic peak in March 2016 (after which half the cases were accrued) [13]. Similar to 2013/14, the current season’s epidemic may have already peaked nationally in Canada; however, there is regional variation in the timing and intensity of activity [1]. Differences in VE estimates at end-of-season analysis cannot be ruled out.

Globally, influenza A(H1N1)pdm09 viruses are in genetic flux, with substantial heterogeneity in circulating clade 6B.1 viruses, but no dominant drift (immunological escape) variant yet declaring a fitness advantage (as in 2015/16) [8]. Consistent with virus characterisation in Europe [7], all sentinel A(H1N1)pdm09 viruses sequenced here belonged to clade 6B.1 and bore additional S74R (Cb), S164T (Sa) and I295V mutations. Across various genetic subgroups, most viruses (83%) also bore S183P. Although the latter is not within an antigenic site, the introduction of proline (a large aromatic ring) in such close proximity to antigenic site Sb could have structural effects. A slim majority of sequenced viruses (57%) in several subgroups bore T185I substitution. Amino acid 185 in antigenic site Sb first mutated during the 2010/11 season and became established in the A(H1N1)pdm09 population as clades 6 and 7, with S185T becoming dominant in subsequent seasons. The extent to which T185I substitution may now instead prevail warrants monitoring, especially when present alongside S183P as prominently identified in western Canada. Of note, antigenic site Sa has also evolved considerably over the past seven seasons (with mutations localised around amino acids 160–163). While the 2013/14 A(H1N1)pdm09 epidemic was dominated by a K163Q mutant and 2015/16 by the clade 6B.1 S162N variant, this season L161I and K160M substitutions have arisen, albeit infrequently (12% and 2% of viruses, respectively). These substitutions may be particularly relevant since they are located adjacent to an important, experimentally determined B-cell epitope [11]. Further, the amino acid at position 74 in site Cb continues to drift, with 4% of SPSN viruses carrying either the R74I or R74G substitution.

That interim VE estimates were not markedly affected by this genetic heterogeneity is consistent with findings from national surveillance systems in Canada, the United States (US) and Europe reporting few influenza A(H1N1)pdm09 viruses (< 5%) manifesting antigenic drift from the A/Michigan/45/2015 vaccine strain [1,7,14,15]. In Canada, low-level antigenic distinction has been restricted to viruses bearing a substitution at position 156, within the immuno-dominant Sa site and previously recognised as influential on antigenicity and receptor-binding properties [16-18]. Mutations at position 156, however, were identified in just 2% of SPSN sequences so far this season. In combination, these findings reinforce the World Health Organization’s decision to retain the A/Michigan/45/2015 strain for the forthcoming 2019 southern hemisphere vaccine [5]. However, global monitoring for further evolution in circulating variants remains important to inform potential vaccine reformulation for subsequent seasons.

As in prior SPSN analyses [2-4], most participants in the current analysis were adults aged 20–64 years (62%). However, 1–8-year-old children appeared to be disproportionately affected, accounting for 28% of A(H1N1)pdm09 cases overall in our outpatient setting (despite comprising ca 9% of the underlying population and 14% of controls across SPSN provinces [19]). This paediatric involvement is also consistent with national surveillance findings for 2018/19 [1] but may be more pronounced in the current analysis than in previous A(H1N1)pdm09-dominated seasons. For example, 160 (30%) of 540 unvaccinated influenza A(H1N1)pdm90 cases in 2018/19 were 1–8-year-old children (Table 1) whereas their corresponding contribution was significantly lower in 2015/16 (32/237; 14%; p < 0.001) and 2013/14 (32/259; 12%; p < 0.001), despite more comparable representation among unvaccinated controls across the same mid-season analyses in 2018/19 (98/623; 16%) compared with 2015/16 (77/454; 17%; p = 0.66) and 2013/14 (37/332; 11%; p = 0.04) [3,4]. Overall, the median age of unvaccinated influenza A(H1N1)pdm09 cases was lower this season (29 years) than in 2015/16 (36 years; p < 0.001) or 2013/14 (35 years; p < 0.001) [3,4]. These age-related differences may reflect a greater proportion of children younger than 9 years in the current epidemic who were not yet born during prior H1 epidemics – notably the 2009 pandemic of nine years ago – with fewer opportunities to acquire immunity compared to older age groups. 

Limitations of the current analysis include its observational design for which residual bias and confounding cannot be ruled out. Sample size was limited in age-stratified analyses, requiring cautious interpretation. In addition, characteristics of the 2018/19 influenza season have varied across the northern hemisphere. While the season in Canada commenced earlier than in recent years [1], notable influenza activity in the US and most of Europe was not observed until mid-December [14,15]. Further, while influenza A(H3N2) has accounted for less than 10% of subtyped A viruses in this study and nationally in Canada [1], influenza A(H3N2) has predominated in south-eastern regions in the US, and co-circulation has been observed in Europe, with influenza A(H3N2) accounting for about one third of subtyped influenza A detections [14,15]. It remains to be seen how varying virological and participant profiles will impact VE estimates elsewhere across the northern hemisphere.

## Conclusions

Interim estimates from Canada for the 2018/19 northern hemisphere indicate substantial VE of ca 70% against influenza A(H1N1)pdm09. Thus far, this epidemic has taken a greater toll on children younger than 9 years even when compared with previous A(H1N1)pdm09-dominant seasons. Given ongoing epidemic activity in some regions, vaccination should be advocated to minimise the A(H1N1)pdm09-associated disease burden. In the context of observed genetic diversity, monitoring for further evolution in circulating 6B.1 variants, and potential impact on vaccine protection, is warranted.
